# Situational analysis of communication of HIV and AIDS information to persons with visual impairment: a case of Kang’onga Production Centre in Ndola, Zambia

**DOI:** 10.1186/s13104-017-2473-4

**Published:** 2017-04-04

**Authors:** Grace Nsangwe Chintende, Doreen Sitali, Charles Michelo, Oliver Mweemba

**Affiliations:** Department of Public Health, School of Public Health, University of Zambia, P.O. Box 50110, Lusaka, Zambia

**Keywords:** Situational analysis, Communication, HIV and AIDS, Information, Visual impairment

## Abstract

**Background:**

Despite the increases in health promotion and educational programs on HIV and AIDS, lack of information and communication on HIV and AIDS for the visually impaired persons continues. The underlying factors that create the information and communication gaps have not been fully explored in Zambia. It is therefore important that, this situational analysis on HIV and AIDS information dissemination to persons with visual impairments at Kang’onga Production Centre in Ndola was conducted. The study commenced in December 2014 to May 2015.

**Methods:**

A qualitative case study design was employed. The study used two focus group discussions with males and females. Each group comprised twelve participants. Eight in-depth interviews involving the visually impaired persons and five key informants working with visually impaired persons were conducted. Data was analysed thematically using NVIVO 8 software. Ethical clearance was sought from Excellency in Research Ethics and Science. Reference Number 2014-May-030.

**Results:**

It was established that most visually impaired people lacked knowledge on the cause, transmission and treatment of HIV and AIDS resulting in misconceptions. It was revealed that health promoters and people working with the visually impaired did not have specific HIV and AIDS information programs in Zambia. Further, it was discovered that the media, information education communication and health education were channels through which the visually impaired accessed HIV and AIDS information. Discrimination, stigma, lack of employment opportunities, funding and poverty were among the many challenges identified which the visually impaired persons faced in accessing HIV and AIDS information. Integration of the visually impaired in HIV and AIDS programs would increase funding for economic empowerment and health promotions in order to improve communication on HIV and AIDS information. The study showed that, the visually impaired persons in Zambia are not catered for in the dissemination of HIV and AIDS information. Available information is not user-friendly because it is in unreadable formats thereby increasing the potential for misinformation and limitations to their access. This calls for innovations in the communication on HIV and AIDS information health promotion to the target groups.

## Background

Blindness and vision impairment are major public health problems causing a substantial human and economic toll on individuals and society including significant suffering, disability, loss of productivity, and diminished quality of life for millions of people. Vision impairment often affects people’s ability to drive, read, learn, watch television, or simply attend to common household or personal tasks [1].

Reduced vision among mature adults result in social isolation, increased risk of falling and resultant hip fractures, depression, family stress and ultimately to a greater extent leads to disability or to premature death. It is commonly perceived that persons with sensory, physical, intellectual and developmental disabilities are not at risk of HIV. There is a mistaken assumption that they are less likely to be sexually active, involved in drug abuse or engage into other risk behaviors [2]. Moreover, national HIV strategic plans, HIV-related services and HIV outreach efforts have overlooked persons with disabilities. Persons with disabilities are not reached by the general HIV prevention, care, support and treatment campaigns and other services [2].

Additionally, because of the many barriers, persons with disabilities often require an escort by a family member or acquaintance in order to access services. They may experience trouble finding escorts due to the fear of being associated with HIV and AIDS. Using escorts also means that persons with disabilities are denied the basic confidentiality when learning their HIV issues [1].

According to Global Health Policy (2014), there were 35.3 million people living with HIV in 2012 from 29.4 million in 2001. In the sub-Saharan Africa, the hardest hit region is home to 71% of the world’s total population of people living with HIV, yet it is only about 12% of the world’s total population. The latest Demographic and Health Survey indicates that, 106,400 (12.7%) Zambians aged between 15 and 49 were living with HIV and AIDS [3].

In the context of HIV and AIDS, persons with disability are at risk just like any other person including commercial sex workers and their clients, injecting drug users, homosexuals, orphans, women and prisoners [4].

World Health Organization (WHO) indicates that, 650 million people representing 10% of the world’s population have a disability. Four out of every five disabled persons live in developing countries [5]. Further, it has been estimated that 4–5% of children who have lost one or both parents due to AIDS also have disabilities across the globe [6]. In Zambia about 15% of the population is disabled [7]. Disability sub-groups are generally identified as the deaf and hard of-hearing, the blind and visually-impaired, people with physical disabilities and people with intellectual impairments or “mental health users [8]. A higher percentage of people with disabilities live in rural areas where access to basic services is limited [7]. Furthermore, an increase in people living with disabilities in many parts of Zambia has been reported [8]. This has been attributed to various factors such as the increase in accidents, congenital disorders, diseases and malnutrition [8].

The global view on the statistics indicates that, at present, little is known about HIV and AIDS and disability. Only a few studies in North-America have estimated a prevalence of 14–15%. No prevalence rate data exist for any populations from Europe, Asia, Central and South America, the Caribbean or sub-Saharan Africa [9]. Reports show that there was no data on the prevalence rate of HIV infection in any disabled populations in Africa. On the other hand, a growing number of disability advocates worldwide point to significant unreported rates of HIV and AIDS related infection, disease and death [2].

Although studies on HIV prevalence among persons with disabilities are still few in Africa, a study recently conducted in South Africa revealed an HIV prevalence rate of 12.5% among sexually abused female adolescents with mental disabilities [10]. Again, a South African national study conducted in 2008 showed an HIV prevalence rate of 14.1% among persons with disabilities which is close to the national prevalence of 16.9% among age group 15–49 years old [10]. Similarly, among the deaf population in Yaoundé in Cameroon, the HIV prevalence rate was 4% similar to the prevalence rate of 4.7% in the city. Over a 2-year period, 7% HIV prevalence was documented in Kenya. These studies serve as proof that Africans with disabilities are surely at risk of HIV infection [11].

Globally, programmes and campaigns to create awareness on how to prevent manage and live positively with HIV and AIDS have rarely been made accessible to visually impaired persons [12]. A notable example is that of factors influencing communication on HIV and AIDS information and related topics which are not available in Braille or large print, hence, inaccessible to the visually impaired people.

It is argued that once infected with HIV and AIDS, visual impaired people are likely to experience reduced support [13].

Despite the increases in health promotion and education programmes on HIV and AIDS, there is still lack of information on HIV and AIDS and communication for persons with visual impairment. The underlying factors that create information and communication gaps on HIV and AIDS have not been fully explored in Zambia [11]. It was therefore, important that this study on communication of HIV and AIDS information to persons with visual impairment at Kang’onga Production Centre in Ndola district was explored [11].

## Methods

### Research approach

A qualitative case study was adopted. The respondents were persons with visual impairments living at Kang’onga Production Centre. The case study was helpful in getting a detailed insight of persons with visual impairments on various views explaining relationships and describing individual experiences. It helped in understanding the perceptions of people working and programmes associated with persons with visual impairments.

### Study site

The study site was Kang’onga Production Centre for the disabled in Ndola rural a quasi-organisation of the Zambia Agency for Persons with Disabilities (ZAPD). It is a public employment Centre for persons with disabilities founded in 1964 as Kang’onga resettlement Centre for the blind and was later changed to Kang’onga Production Centre. It used to accommodate other types of disabilities though presently the visually impaired are the majority residents. The Centre manufactures brushes, rattan (cane) furniture, rattan baskets and trays. This study site was purposively selected because it has a reproductive population of the visually impaired persons and it is the only production Centre for the visually impaired in the country.

### Study participants

The respondents included persons above 18 years of age comprising the visually impaired residing at the Centre, personnel working with persons with visual impairments, health education and promotion staff at the District Medical Office (MDO), staff at the National AIDS Council in charge of health information, staff at the Zambia Library for persons with visual impairments and the manager at Kang’onga Production Centre.

### Sampling and sample size

Maximum variation sampling was used until optimal theoretical saturation was reached for the visually impaired persons. This type of sampling, involved searching for cases or individuals who met a certain criterion. Thus, the visually impaired and residing at Kang’onga Production Centre were this criterion. It involved equal representation of both men and women. There were differences in age, gender, marital status and education in four categories of those above 50 years and those below 50 years to appreciate the experience. The sample size comprised two focus group discussions and twelve in-depth interviews.

The second sampling method used was the expert availability sampling in order to enlist key informants or individuals working with persons with visual impairments at ZAPD staff at the DMO and at the National AIDS Council in charge of health information, staff at the Zambia library for persons with visual impairments and the manager at Kang’onga Production Centre. Expert availability sampling strategy is particularly useful in the context of a situational and policy analysis. This sampling strategy involves identification of major stakeholders involved in designing, giving, receiving or administering the programmes or services and those might otherwise be effected or be affected.

### Data collection

In-depth interviews and focus group discussions using the semi-structured interview guides were used to collect data from different kinds of informants in a form of triangulation. ‘Data triangulation’ is used to contrast the data and ‘validate’ the data to establish where it yields similar findings [14].

### In-depth interviews

Eight in-depth interviews (IDIs) with the visually impaired persons were conducted under four categories of gender, marital status, education and age. These categories were used in order to give an in-depth understanding of different categories of individuals. An interview schedule was used. The interviews were conducted at a place that was comfortable to the respondent using a digital recorder to record the interviews with permission from the participants.

### Focus group discussions

Two focus group discussions (FGDs) were held with persons with visual impairments. One FGD was with the women and the other with the men. Each FGD had twelve participants and two facilitators, one moderator while the other was taking notes and recording. The FGDs were audio recorded.

### Key informant interviews

Data were collected using Key informant interviews, in-depth interviews and focus group discussions. Key informant interviews were conducted with people who knew what was going on and who could have been involved in programming or developing interventions for the visually impaired such as individuals working with persons with visual impairments at ZAPD; health education and promotion staff at the DMO; Staff at the National AIDS Council in charge of health information; staff at the Zambia Library for persons with visual impairment and the manager at Kang’onga Production Centre. The key informants were selected because of their specialised knowledge and unique perceptions on the topic under study. This was to collect information from a wide range of people with first-hand knowledge about the community and experts in understanding and able to provide insight on the nature of problems and able to recommend solutions.

### Data processing and analysis

Data processing and organization was done immediately after each interview in order to ensure that the interviews notes and participants were properly labeled for easy management. Audio files were also marked with codes together with all notes taken during the interviews whose duration was not more than 60 min per interview. Names were not included to avoid linking these to any respondent. A verbatim transcription was done. Interviews in local languages were translated into English.

Data were analysed thematically and themes were created. The initial coding framework for the collected data generated to code data while new codes were created for emerging themes as more data was reviewed. NVIVO 8 programme software was used to analyze the data which also helped in the management of the coding process. Sub-themes, categories and themes that were generated after analysis of the collected data are shown in Table 1.

**Table 1:** **Table showing sub-themes, categories and themes**

**Sub-themes**	**Categories**	**Themes**
Misperception of cause	Cause	Knowledge on HIV/AIDS
Unprotected sexual intercourse, sharp instruments, use of traditional herbs	Transmission	
Protected sex through the correct use of condoms, abstinence, being faithful to your partner	Prevention	
Radio, television, Public announcement systems, drama	Media	Ways through which the VI access information
Charts, braille books, HIV/AIDS Education, Pamphlets		
Peer education	IEC materials	
Health centers visits, specialized staff, disability specific, Community home based care and Anti-natal	Health education	
Workshops, Counseling, health education		
Asexual claims by the sighted, difficult people to teach		Challenges the VI face in accessing HIV/AIDS information
Double discrimination, cultural barrier and acceptance	Discrimination/Stigma	
Illiteracy among the VI, Lack of IEC’s materials, No Programs specifically for VI	Education	
No Access to radio, easily taken advantage, distance	Poverty	
Structures do not integrate the VI, Challenge in isolating and Mobilizing special groups, Not accommodated in decision making	Integration	
Few VI in employment	Employment	
Poor funding, discriminatory funding, low staffing	Funding	
Government to stop neglecting persons with VI, to be involved in planning	Integrate	Promotion of communication of HIV/AIDS information for persons with VI
Equal participation in HIV/AIDS mainstream interventions		
Representation of the VI in all structures		
Government to Increase funding and source solar radios, initiate economic empowerment programs	Funding	
Increase awareness, train health professions on the needs of the VI	Health promotion	
Translation of HIV/AIDS information in readable and local languages, audio recordings		
Entertainment for the VI collaboration between line ministries and other agencies		

## Results

### Introduction

The findings of this study are structured under the themes derived from the research questions. The four research themes are knowledge on HIV and AIDS, ways in which visually impaired persons access HIV and AIDS information, challenges visually impaired persons face in accessing information on HIV and AIDS and promotion of HIV and AIDS information among the visually impaired persons.

### Socio-demographic characteristics

Participants’ age range in the study was between 18 and 60 years old out of which thirty-two were persons with visual impairments and the remaining five were key informants working with the visually impaired and health promoters. The majority of the respondents were aged between 30 and 42 years.

### Attributes of persons with visual impairments

The visually impaired participants had varying attributes of age, being an indigenous resident of the Kang’onga Production Centre and education (ability to read Braille and not able to read). The other attribute was gender because both males and females were included. The other attribute was marital status since both married and single respondents were identified.

### Knowledge on HIV and AIDS

Respondents when asked to explain what they knew about HIV and AIDS. The responses obtained revealed that there were variations among them regarding knowledge on HIV and AIDS. Some respondents were aware while others had limited awareness in the cause, transmission, prevention and had misconceptions. On the other hand, the respondents exhibited knowledge on treatment of HIV and AIDS. Following below are results of what the visually impaired persons say they know about HIV and AIDS and what their experiences are.

### Perceived causes and transmission of HIV and AIDS

The findings on the perceived cause of HIV and AIDS included some perceptions that HIV and AIDS came from urine, wounds, coughing, diarrhea, tuberculosis and from the laboratory to kill the black race as testified by one male respondent in the age range of 25–32 years who said:

HIV and AIDS were made in the laboratory to kill the black race

Further, despite other participants’ revelations of the mode of transmission of HIV and AIDS, some participants interviewed attested that HIV and AIDS came through some diseases such as coughing and fever as another respondent alluded that:

HIV and AIDS came through coughing, fever. When one has these symptoms they should see the doctors because HIV and AIDS came such diseases.

In terms of the knowledge on the transmission of HIV, most of participants said one can get infected through sexual intercourse. Some participants also stressed that, HIV transmission occurred when one engaged in unprotected sexual intercourse as one respondent stated that:

HIV and AIDS come through unprotected sexual intercourse with a person who is positive and using razor blades after someone has used it.

Information from in-depth interviews revealed other ways perceived by the visually impaired persons how HIV was transmitted such as razor blades, during child delivery and injections. Some participants also indicated that women used traditional medicines to make their vaginas dry to increase sexual pleasure for the men. This causes bruises during sexual encounters and the friction led to HIV transmission. This was explained by one married male participant in the age range of 30–40 years when he said:

Even through injections that we get from the hospitals if they inject one who is HIV positive. Nurses when they are delivering during labour, if treatment is not favourable that person assisting if he or she has no gloves for protection can be infected if someone is not following instructions we are given when having sex such as the use of condoms and women not to be using herbs for dry sex. Dry sex makes people to have bruises during sexual intercourse.

### Treatment and prevention

Related to treatment and prevention, it was reported that there was no cure for HIV and AIDS but rather medicines to prolong life were there as one participant put it that:

We know that when you are found HIV positive, you can be given medicines to prolong your life … I was told, you cannot be healed, you can just be helped how to live long with the disease.

In terms of prevention, most participants indicated that also it was a known fact that there was no cure for HIV and AIDS and as such they said the only way was to prevent HIV and AIDS by being faithful to their partners, using of condoms, abstinence and avoiding sharing of blades. However, some female participants in the in-depth interviews argued that, they did not trust a condom because they could not see if it was being used correctly or if a partner had put a hole in it. This was stressed by one female respondent when she said:

Using and trusting a condom is a challenge, because one cannot know if a partner was using it correctly or had ripped it and besides we hear that a condom is not safe.

However, some male participants argued that, even if they were visually impaired they were able to use a condom correctly because they were also human beings as testified below by one male respondent when he said:

Even if we cannot see, we also know how to use the condoms correctly just like those who see.

On the overall, the visually impaired persons showed a variation on the HIV and AIDS knowledge because of the different responses on the cause, transmission, treatment and prevention.

### Ways in which the visually impaired persons access HIV and AIDS information

Participants indicated a number of ways through which persons with visual impairments became aware of HIV and AIDS. These included the Media, information education communication materials (IECs) and health education.

#### Media

Information from both the in-depth interviews and focus group discussions indicated that most participants had common ways in which they accessed information on HIV and AIDS which included listening to the radio, television, public address systems and drama or plays and sketches. This was affirmed by what one female participant who said:

We gain HIV and AIDS information by listening to those who teach about HIV and AIDS and listening to the radio and television because they also teach about HIV and AIDS.

Drama as an expressive art is another critical way through which people transmitted information. This mode is also used to communicate information on HIV and AIDS to the visually impaired persons. Drama was cited by most participants as a preferred way of accessing information on HIV and AIDS. One female participant from FGD of age range of 38–42 years argued that:

Even through sketches that teach on how one can take ARVs and how HIV and AIDS is transmitted from one person to another would be used to access HIV and AIDS information.

Further, some key informants involved in health promotions revealed that when mobilising people for HIV and AIDS programmes, they use the public address system (PA) and said:

Currently, we have the public address (PA) system. Using big speakers while going round the community making announcements on health issues to the public.

#### Information, education and communication (IEC) materials

The other channels through which the visually impaired received information established from the responses especially from those who were able to read Braille were through books transcribed into Braille. However, the participants noted that Braille was hardly accessible as the importance of Braille was illustrated by one male participant who observed that:

Through health education on HIV and AIDS, Braille books though unavailable … the best way to educate us on HIV and AIDS information is through books in Braille.

Pamphlets were sources of written information for persons with visual impairment and it was pointed out as one of the ways HIV and AIDS information was accessed.

Even through books, we have pamphlets and Braille which talks about HIV and AIDS.

It was also learnt from the key informants or health promoters that charts were among the ways information is given to persons with visual impairments though these were not specifically designed for them. These charts had HIV and AIDS messages and other health concerns which were of help to both the sighted and the visually impaired. One key informant echoed these sentiments when he said:

We have the charts with different important messages that we need to communicate but the health education we give is the same whether you are a visually impaired person or you are able person.

#### Health education

Most participants revealed that one of the major ways they accessed HIV and AIDS information was through health education. They cited visiting health centres, trainings, peer education workshops; community home based care and counseling as some of the ways through which they accessed information on HIV and AIDS.

Peer education and Health Centre visits were another way through which the visually impaired persons accessed information. However, it was also observed that the visually impaired did not usually visit health centres specifically for HIV and AIDS but for other health ailments. It was during such visits that sometimes they coincidentally accessed HIV and AIDS information. The statement below attests to one female participant’s views that:

By listening to those who teach about HIV and AIDS, listening to the radio and television because they also teach about HIV and AIDS. Visiting clinics and hospitals, because each time we go there they sometimes teach us on HIV and AIDS. I also escorted my friend to a clinic and was taught about HIV and AIDS.

The other way female participants accessed HIV and AIDS information was through workshops, community home-based care, counselling and antenatal visits. Participants argued that it was during such programmes that they received HIV and AIDS information and shared information with each another. However, despite these methods being used, the visually impaired persons stated that they were rarely invited to attend these programmes. Hence most participants argued that the best way of communicating HIV and AIDS information to the visually impaired persons was through the one-on-one teaching and demonstrations. The two sentiments that reflect this argument are that:

Through workshops, community home based care and Anti natal they hear about HIV and AIDS and counselling from the clinic, though not specifically but the general one.

Therefore, participants indicated a number of ways through which persons with visual impairments received HIV and AIDS. They revealed ways such as the media, information education communication materials (IEC) and health education.

### Challenges faced by the visually impaired persons in accessing HIV and AIDS information

When asked on the challenges that persons with visual impairments face in accessing HIV and AIDS information, participants highlighted a number of ways which included discrimination and stigma, education, poverty, integration, employment and funding.

#### Discrimination and stigma

Discrimination and stigma seemed to be a big challenge among the visually impaired persons. Some of the participants felt they were discriminated against because information on HIV and AIDS rarely reached them and as such, the visually impaired persons assumed that they were considered less compared to their sighted counterparts. One of the participants in the in-depth interview observed that double discrimination was also a challenge because those who were found to be HIV and AIDS positive would even be neglected by e their own families. These challenges were attested to in the quotes cited below:

Most people involved in the disseminating of HIV and AIDS information did not come to us regularly on HIV and AIDS; they don’t come to us because they mostly think we don’t get sick. They should know that we also get sick. They consider us less when giving us information.

When I was found to be HIV positive, my sighted husband left me and my family chased me from home and I went to stay with friends. They told me that I was going to die and that they do not have money to buy a coffin.

#### Education

The level of education was identified as having a bearing e on HIV and AIDS information dissemination to persons with visual impairments. For example, most of the visually impaired persons were unable to read and write Braille. The participants reported that most HIV and AIDS programs were in English which they did not understand. According to the respondents interviewed, they argued that they were not cared for because much concentration and care was directed to the sighted persons. It was revealed that they had challenges on the escorts (the people who took and guided them from one place to the other), hence they found it difficult to visit health centres. Even when they managed to visit clinics, they remained unattended to for a long time as one female respondent indicated when she observed that:

Most HIV and AIDS programs are in English but most of us do not know English and we are not cared for because we are visually impaired. They concentrate on those that can see while we are not frequently given a chance to visit clinics, because people who take us at times refuse. Also at clinics they also discriminate us, you can remain at the clinic unattended to for a long time since we cannot see. Other languages should be used because not everyone knows English.

The study also found out that, the visually impaired persons had no HIV and AIDS messages designed specifically to meet their communication needs. The key informants working with persons with visual impairment confirmed that they did not have programmes tailored to meet the diverse needs of the visually impaired persons.

Yes information is not prepared specifically for the visually impaired persons and we do not have much Braille information.

#### Poverty and unemployment

Besides discrimination, stigma and education, poverty and unemployment were some of the key challenges faced by persons with visually impairments in accessing HIV and AIDS information. It was noted that most persons with visual impairment live in poverty. As such, the majority of them were unable to access health centres due to lack of transport money because they needed to pay for the escort and themselves. It was also noted that despite the radio being the most preferred way of accessing information, few hardly afforded a radio due to poverty. It came out in interviews with participants and key informants that:

Me I have a radio but some of my friends do not have, even though I have this radio cannot always afford to buy cells so sometimes I do not even play the radio and hence miss most of the information.

In the same interview participants argued that, they were more vulnerable to HIV and AIDS infection because they have little or no say on whom to have sex with and how to have it and as such it was difficult for one to prevent HIV and AIDS. One female participant narrated that:

We have no say on whom to have sex because we do not have employment and yet we need to feed so any man offering to help we cannot stop him and we also depend on others to see for us and tell us that he is a good a man and that is how we end up with HIV positive men.

#### Logistical challenges

Some key informants interviewed also said distance was a challenge for them to reach out to persons with visual impairments on HIV and AIDS information. This was not only a challenge for the health promoters to reach out to the visually impaired persons but to the entire population in rural areas. The contributing factor to this is lack of a utility vehicle.

We as are a regional office we do not have a utility vehicle. So even if they say can we meet at Kang’onga today, it will need me to write a letter to the provincial cabinet that I need to do this and I am asking for a vehicle and driver and will pay for fuel and lunch for the driver. It is a challenge to manage a province without a utility vehicle.

#### Integration

Through the focus group discussion for both males and females, integration was cited by the majority that it was another challenge that they faced in accessing HIV and AIDS information. They said, some infrastructures had no lift, wide doors and or ramps thus inhibiting them from accessing HIV and AIDS information as most they do not accommodate persons with visual impairments. This made them fail to access health services on HIV and AIDS especially in hospitals were they were referred to.

Other structures do not integrate the visually impaired in activities despite being documented in the national Act for persons with disabilities, and it is not fully implemented.

The other participants felt not integrated in decision making. As one participant argued that the visually impaired persons or disabled in general did not sit on boards which made decisions to give advice on HIV and AIDS or integrate the visually impaired persons into the HIV and AIDS plans as alluded to by one ZAPD participant when he said:

Acts actually require that whatever you do or plan must integrate people with disability. Even if it’s a national HIV and AIDS plan, or anything to do with building or vaccines one needed to consult on how to integrate the disabled but mostly these guidelines are not followed.

Health promoters stated that the other challenge they faced was in isolating and mobilising special groups to disseminate HIV and AIDS information among the visually impaired persons. This was because there were no specialists to help in isolating special groups such as the visually impaired persons to plan specific communication programmes on HIV and AIDS.

We usually have a challenge when it comes to isolating these special groups as per say… we have no qualified health promoters, even me am not qualified but am in charge of health promotion in this district.

### Improving communication on HIV and AIDS information among the visually impaired persons

In view of the challenges indicated, a number of interventions were suggested on how communication of HIV and AIDS information to and among the visually impaired persons could be improved and enhanced. These included improving integration, funding and awareness and sensitisation. It was observed that a lot of things had to be put in place. It was revealed that paramount to accessing HIV and AIDS was through integration of the visually impaired persons into HIV and AIDS programmes and programming. Views were expressed by a young participant in the age range of 20–25 years that:

The abled should consider as persons with sexual feelings and not discriminate us from HIV and AIDS programmes and we should also be taken to radio stations to teach others on HIV and AIDS. They should integrate us.

Participants cited trainings, workshops and mainstreaming as tools in the promotion of communication on HIV and AIDS information. They said training the visually impaired persons would enable them to become peer educators. Involvement in programmes such as HIV and AIDS programming and workshops would enhance their HIV and AIDS information accessibility. Readable formats for the visually impaired persons should be availed to those who were able to read because they can become the mouthpiece for the other visually impaired persons who are unable to read. This was supported by a married participant in age range of 30–40 years old who said:

They should be training us so that we can also teach others on HIV and AIDS and we should be encouraged to teach other. Also we should be integrated in workshops, trainings and planning. Braille books should be made available to us who can read and write.

### Funding

Prominent in the recommendations was the need for the government to stop neglecting the visually impaired persons in funding. They argued that most funds on HIV and AIDS went to programmes that benefited the sighted which was wrong as they were also affected and infected by HIV and AIDS. They argued that funds should be made available for programmes that benefited persons with visual impairments in accessing HIV and AIDS information as indicated by some participants when they said:

Us who have challenges of sight, the key people to promote or overcome these challenges that we face is government by stopping neglecting us and also to include the blind in HIV funds because mostly government releases funds for HIV and AIDS.

Entertainment was also advanced as one key area that needed to be taken care of if information on HIV and AIDS was to be prioritised among the visually impaired persons. It was revealed that drama or sketches and plays could be enhanced especially for those who cannot read or afford a radio to learn something on HIV and AIDS. In the same line, also it was indicated that there was need to include sporting activities because they lack such social amenities. As noted by people working with the visually impaired persons embraced sexual intercourse as the greatest means of entertainment as pointed out that:

Not that we are trying to make a story sweet but it is a fact they do not have any other entertainment that’s why the biggest entertainment they have is sexual intercourse so why not create awareness.

Collaboration between ministries and other agencies was also mentioned. In order to serve the visually impaired persons better in terms of communication on HIV and AIDS information, it was argued that there was need for collaboration between ministries and other agencies.

Ministries have to come together like in the old days to meet the people and give them hand-outs. We can design and make a programme for that. So that even in churches they can be reached though not in the majority but as a fraction for we know that the visually impaired persons are everywhere

The need for an escort was another challenge which needed to be worked on the accessibility of HIV and AIDS information. It was argued that usually they needed a child to guide or escort them and yet the child had also to go to school. Meaning that, the visually impaired person would have no one to escort them thereby not accessing information or treatment at the health facility. Therefore, it was suggested by the female FGD that it would be better for the hospital to help the visually impaired person. A female participant in the age range of 35–41 years old complained that:

They know the dates they conduct anti-retro therapy (treatment) so if they can send those people they call caregivers to come and take us because most times we are asked that why have you come alone and no one to lead you. Ministry of health can help us by sending caregivers to come and be our escorts especially on the dates for attending ART clinics so that our children can attend school as well.

## Discussion

### Introduction

The study aimed at understanding the communication of HIV and AIDS information for persons with visual impairments. A general overview and discussion of results in comparison and contrast with other studies on the four research questions has given the limitations of the study.

### Summary of results

It was evident from this research that there was a variation in terms of knowledge on HIV and AIDS among persons with visual impairments in the cause, transmission, treatment and prevention of HIV and AIDS. It was established that there were a number of ways in which persons with visual impairment accessed information on HIV and AIDS. These included the media, IECs and health education. In terms of the challenges in accessing HIV and AIDS information, it was found out that the visually impaired persons experienced discrimination, stigma, illiteracy, inadequate IEC materials, lack of information specific programmes, inadequate specialists to mainstream the planning of specific communication programmes on HIV and AIDS for the disabled, poor integration of the visually impaired persons in decision making processes, poverty and logistical and funding difficulties to implement programmes.

To counter the identified challenges, it was recommended that there should be increased integration of the visually impaired persons into HIV and AIDS programmes and programming, training, workshops, peer education and as well as development of appropriate readable formats of the required literature. An appeal was made for increased funding for programmes that did not only economically empower the visually impaired but those that would increase capacity for programme managers to develop awareness and sensitisation programmes on HIV and AIDS in appropriate formats and language for the visually impaired.

### Knowledge on HIV and AIDS

There was a knowledge gap on HIV and AIDS among the visually impaired persons. This knowledge gap, it was established to be in different critical domains of the cause, transmission, treatment and prevention. Little knowledge on the cause of HIV and AIDS was indicated as a result of other diseases. On the mode of transmission, an understanding showed that there were two modes, thus through unprotected sexual intercourse and the use of sharp instruments such as razor blades and syringes.

It was established that there was substantial knowledge on the treatment and prevention while to some extent there was inadequate information on HIV and AIDS. It was also highlighted that HIV and AIDS had no cure and that could be prevented through the use of condoms during sexual intercourse, abstaining and being faithful to one sexual partner. This is in agreement with other findings from studies conducted in other sub-Saharan African countries [15].

Literate visually impaired persons showed more awareness and clear knowledge on HIV and AIDS compared to their illiterate counterparts. The low literate levels disadvantaged the visually impaired and other disabled persons [2, 16].

### Ways in which the visually impaired access HIV and AIDS information

There are many channels through which persons with visual impairments access HIV and AIDS information, some of which are the media, information, education and communication (IEC) materials and health education. Other studies conducted across sub-Saharan countries, for example, in Malawi, a study on *‘Effective HIV and AIDS and Reproductive Health Information to People with Disabilities’*, revealed that people with visual impairments mostly communicated with others through speech because they were able to talk and hear. It was demonstrated that radio; television, drama, Braille, large print and electronic media constituted some of the common channels of communicating HIV and AIDS messages to the visually impaired persons [17].

In contrast, although antenatal clinics were cited as one way through which female visually impaired persons accessed information on HIV and AIDS, other study findings indicated that females with disabilities faced numerous physical and attitudinal barriers to accessing fertility and antenatal care [2].

It was established that there were no trained staff or experts to attend to the communication needs of the visually impaired persons on HIV and AIDS in organisations and health institutions. This is why most channels are not fully utilised by health promoters to disseminate HIV and AIDS information to persons with visual impairments.

### Challenges people with visual impairment encounter in accessing HIV and AIDS information

Despite the visually impaired persons accessing information on HIV and AIDS, there are many challenges faced. The challenges revealed among others were discrimination and stigma. It was established that the visually impaired persons were discriminated against and stigmatised when accessing information on account of being visually impaired. This could be attributed to the unavailable appropriate formats for HIV and AIDS information. In the same vein, a study conducted on *“Perceptions of the Availability and Effectiveness of HIV and AIDS Awareness and Intervention Programmes by People with Disabilities in Uganda”* also found out that the visually impaired persons felt discriminated against on HIV and AIDS issues because they had difficulties in accessing HIV and AIDS services because of mainly communication problems [16].

One of the other challenges was that the visually impaired found to be HIV and AIDS positive faced double discrimination because their own families and health practitioners discriminated against them. Similar findings were also reported by other scholars that HIV and AIDS-related stigma and discrimination were a major challenge for persons with visual impairments because it affected the individual, the family and the society at as a whole [18].

The level of educational attainment for persons with visual impairment was another challenge that affected the access to information on HIV and AIDS. Many visually impaired persons are illiterate and have misconceptions on the cause, transmission and treatment of HIV and AIDS. These findings are in line with those of the Zambia Federation of the Disabled (ZAFOD) which stated that most persons living with disability, especially the visually impaired, did not have access to information through the radio, television and newspapers or other literature due to the high illiterate levels. This was caused mainly by discriminatory funding by government. It was also indicated that the under-staffing led to failure to carry out effective HIV and AIDS communication programmes for persons living with visual impairments [19].

Further, it was discovered that there were no IEC materials for persons with visual impairments in delivering HIV and AIDS information. People who designed materials on HIV and AIDS mainly think that persons with visual impairments cannot engage in risky sexual behaviours. This is why there were no Braille materials, large print, charts or pamphlets for persons with visual impairments on HIV and AIDS information. This affirmed similar findings in other studies which noted that, IEC interventions were used to alert the general public about the risks of HIV and AIDS were based on the assumptions that HIV and AIDS knowledge would cause change in people’s sexual behaviours from risky sexual behaviours to non-risky behaviours or even safer sex practices. It was explained that HIV and AIDS education on the transmission and preventive measures however had not particularly targeted the visually impaired [20].

The radio was a key medium through which the visually impaired persons accessed HIV and AIDS information because it was able to benefit both those who can read and those who are unable to read Braille. However, in contrast to this, it was also established that, many visually impaired people were unable to afford radios or pay the electricity bills or to buy batteries. Therefore radio campaigns cannot reach many visually impaired people. In most cases also because there were no programmes tailored to meet their communication needs. Worse still, it was also cited that the radio reception of some radio stations were poor in Kang’onga area. This was in contrast with other findings from studies conducted across which showed that the most common medium for providing information about HIV and AIDS in sub-Saharan Africa was through radio campaigns. Radios are more accessible to some people living in rural areas and are more affordable than television sets [2, 17, 18].

Health promoters revealed that the lack of specific programmes for the disabled and specialists to isolate special groups such as the visually impaired persons was a challenge in planning for specific communication programmes on HIV and AIDS. These findings are in affirmation with other research findings conducted in several other countries such as Ethiopia, Kenya, South Africa, Mozambique, Rwanda and Uganda which have shown that many health practitioners lack the necessary skills to adequately respond to the needs of persons with visual impairments [21].

Poverty and distance were the other challenges noted that affected the majority of persons with visual impairments. These resulted in inability to afford basic necessities which included access to referral health centers due to transport challenges as they were supposed to pay for two people—the escort and themselves. These findings are not in isolation because other studies have also indicated that the majority of individuals who were visually impaired in other developing countries failed to meet direct and indirect costs of financing their health care [17].

### Improvement of communication on HIV and AIDS Information among the visually impaired

In order to improve the communication on HIV and AIDS information among the visually impaired persons, a number of interventions could be promoted. Prioritizing mass media campaigns, social marketing and skills development were recommended integrate persons with visual impairments into HIV and AIDS programmes and programming. This would enable equal representation of persons with visual impairments and give them opportunities to be empowered with information on HIV and AIDS. These findings from the study are in line with the United Nations report (2006) which stated that, people with disabilities are in need of the same HIV and AIDS information, services and support just like all other members of society. They can no longer be an afterthought as this corresponded with the United Nations Convention on the Rights of Persons with Disabilities (UNCRPD) [22, 23].

## Limitations and strength of the study

The first limitation was that one group of visually impaired were sampled based on purposive sampling which also cannot be generalised. The lived experiences expressed through verbatim reports cannot be captured by a survey questionnaire. However, for such a study, this is the most appropriate methodology especially when the researcher wants to transfer what was learnt to another setting. The use of maximum variation sampling had the value of representation which was seen in randomization. The researcher being a specialist in special education and as a counsellor enabled for the probes for in-depth information regarding the research topic from both sexes.

## Conclusion

In conclusion, it can be stated that there were variations regarding knowledge concerning HIV and AIDS among persons with visual impairments. The study further showed that persons with visual impairment had no specific materials through which they could access HIV and AIDS information. Health promoters and persons working with the visually impaired persons also faced challenges in delivering HIV and AIDS information to the visually impaired as they lack funding and skilled manpower to reach out to the visually impaired persons in the appropriate formats or modes. It was established that there was an emphasised need for in-depth, context-specific understanding of the challenges in order to promote and improve accessibility of HIV and AIDS formats for persons with visual impairments. Also paramount was the need to implement the development of IEC materials that were disability specific. This is because, HIV risks will continue to scale-up among the persons with visual impairments, and the lack of information may just undermine the gains being scored in scaling-down of HIV and AIDS in Zambia.
